# Central auditory system assessment in children and adolescents with cystic fibrosis: electrophysiology and central auditory processing

**DOI:** 10.1016/j.jped.2024.06.013

**Published:** 2024-09-05

**Authors:** Paula Maria Martins-Duarte, Gabriel Hessel, Antônio Fernando Ribeiro, Fernando Augusto Lima Marson, Maria Francisca Colella-Santos

**Affiliations:** Centro de Investigação em Pediatria da Faculdade de Ciências Médicas da Universidade Estadual de Campinas (UNICAMP), Campinas, SP, Brazil

**Keywords:** Hearing, Auditory pathways, Central auditory diseases, Language development disorders

## Abstract

**Objective:**

This study aims to evaluate the central auditory system of children and adolescents with cystic fibrosis through behavioral assessment of central auditory processing and electrophysiological tests to investigate short and long-latency auditory potentials, comparing them with the results obtained in the control group.

**Methods:**

117 from 7 to 21 years old patients were evaluated, 57 of them with cystic fibrosis and 60 of the control group, using behavioral evaluation of central auditory processing, auditory brainstem response and long latency auditory evoked potential. The comparison of the research groups was performed using ANOVA for Auditory Brain Response and P300 responses and Wilcoxon and Mann-Whitney tests for Central Auditory Processing responses.

**Results:**

A statistically significant difference was found in the results of the GIN test between the groups and in the auditory brainstem response latency responses in waves I and V in the comparison between the groups with higher latencies in the study group. A difference was also found between latencies in the interpeak intervals I-III and III-V. The long latency auditory evoked potential analysis shows a statistically significant difference in the latency of the P300 potential, with higher latencies in the study group.

**Conclusion:**

Cystic fibrosis participants presented worse performance in the gaps-in-noise test compared to the control group in the evaluation of central auditory processing, which indicates impairment of temporal resolution auditory ability. They also showed increased latency in I and V waves of auditory brainstem response, as well as an increase P300 latency in long latency auditory evoked potential.

## Introduction

Cystic fibrosis (CF) is an autosomal recessive disease that affects the exocrine glands. These glands, when producing viscous secretions cause manifestations, mainly respiratory and digestive.^1^

The secretions produced are abnormally viscous, due to dehydration caused by the malfunction or lack of activity of the cystic fibrosis transmembrane conductance regulator (CFTR) protein. It is known that CFTR is encoded by a gene located on chromosome 7 (7q31) and the mutation of this gene causes abnormalities in its functioning. CFTR functions as a chlorine channel, regulating the transport of ions across the cell membrane.^2^ Thus, if there is a deficiency in its functioning, there will be a reduction in chlorine excretion and an increase in intracellular electronegativity, which results in a greater flow of sodium and water. The viscosity of the secretions favors obstruction of the ducts and causes inflammation and fibrosis.^3^

The incidence of CF varies according to ethnicity, occurring more commonly in Caucasians. The incidence is 1 in every 2,600 births in the United Kingdom, 1 in every 2,835 in the United States, and 1 in every 6,902 in Brazil.^4^

The diagnosis of CF is carried out by combining neonatal screening, clinical history and sweat testing (the gold standard in diagnosis), in addition to genetic research.^5^

The microorganisms most commonly found in CF are Staphylococcus aureus, Haemophilus influenzae, Pseudomonas aeruginosa, and members of the Burkholderia cepacian complex.^1^

As colonization by bacteria is frequent in individuals with the disease, the use of aminoglycoside antibiotics is common in the treatment.^6^ One of the effects related to the treatment is the impact of the administration of these drugs on the peripheral auditory system since their ototoxic action on the structures of the inner ear is widely known.^7^ Ototoxic drugs can damage the organ of Corti, degenerating the outer hair cells and may extend to the inner hair cells. First, it can damage the spirals at the base of the cochlea and spread toward its apex,^8^ therefore audiometric tests that assess high frequencies are especially used to detect ototoxicity-related sensorineural hearing loss.^9^

When the ototoxicity of these drugs causes hearing loss, its incidence can vary from 0 to 63%, and in most cases, the damage to the auditory system is irreversible.^10^^,^^11^

The literature presents different studies that used high-frequency audiometry (HFA) to evaluate the onset of ototoxicity at the base of the cochlea.^12^^,^^13^ Fausti et al.^14^ found hearing loss in 47% of the 106 ears evaluated, and in 71% of them, the detection was made by HFA. Mulheran et al.^15^ observed a reduced number of ototoxicities in cystic fibrosis and suggested that the disease may attenuate the progression of ototoxicity due to the rapid renal elimination of drugs and confirmed the absence of ototoxixity through HFA. Cheng et al.^7^ found hearing loss on pure tone audiometry in 14% of patients evaluated after using ten or more aminoglycoside cycles. In another study, Mulheran et al.^16^ used distortion product otoacoustic emissions (DPOAE) to assess hearing and reported that it is more sensitive than pure tone audiometry to identify hearing loss in the early stages. Other authors reinforce that HFA is more effective than DPOAE in identifying initial hearing changes in ototoxicity.^17^^,^^18^

With scientific advances and the development of drugs and treatments for the disease, the authors have observed an increase in the life expectancy of patients with cystic fibrosis and, therefore, greater possibilities of exposure to the ototoxic effects of drugs.

Naturally, for adequate sound stimulation in the auditory system, it is essential that its peripheral structures are intact, thus enabling the transmission of auditory information to the central auditory pathways, up to the region of the auditory cortex. The central portion of the auditory system plays a key role in understanding sound information. Its structures are responsible for the analysis and interpretation of sound information, enabling certain auditory skills essential for language development and learning. If there is a reduction in the ability to capture environmental sound, there may be a change in the organization patterns of auditory skills, contributing to language difficulties and changes in central auditory processing.

Based on the literature, the impacts of the disease itself and its treatment on the central auditory pathways and on the development of central auditory processing skills are not known. Thus, this study aims to evaluate the central auditory system of children and adolescents with cystic fibrosis through behavioral assessment of central auditory processing and electrophysiological tests to investigate short and long-latency auditory potentials, comparing them with the results obtained in the control group.

## Methods

This study was approved by the ethics committee (approval number: 1.421.725) and involved a sample of 117 children and adolescents: 63 females and 54 males, aged between 7 and 21 years (average 13.7 years).

The Study Group (SG) was made up of 57 children and adolescents (minimum age of 7 years and maximum of 21 years, media 14.35, and standard deviation 4.34) diagnosed with cystic fibrosis. The Control Group (CG) was formed by 60 children and adolescents without the disease (minimum age of 7 years and maximum of 21 years, media 13.26, and standard deviation 3.87).

The inclusion criteria for the study group were having a diagnosis of cystic fibrosis confirmed by two sweat tests with a result > 60 mmol/L according to the Cystic Fibrosis Foundation criteria.^19^ The control group should not have any hearing complaints and should not have used aminoglycoside. Participants in both groups should have an intact tympanic membrane visible at meatoscopy and type A tympanometry with compliance between 0.3 and 1.6ml and a peak from the range between -100 and +100daBP.

The study included a preliminary stage with an assessment of the peripheral portion of the hearing. The evaluation was performed using the following procedures: pure tone audiometry (250 to 8,000Hz), high-frequency audiometry (9,000 to 16,000Hz), logoaudiometry and immittance test. The normality criteria considered for pure tone audiometry were those established by the World Health Organization,^20^ for tympanometry the classification of Jerger, Jerger and Mauldin,^21^ and for acoustic reflexes, the classification of Jerger and Jerger.^22^ Logoaudiometry is one of the stages of basic peripheral audiological assessment. It evaluates the speech recognition index and threshold, in order to corroborate the findings in the auditory thresholds. Peripheral and central auditory assessments were carried out in two sessions with a maximum of 20 days between them. Tympanometry was performed at each session.

The behavioral assessment of central auditory processing (CAP) consisted of tests that used verbal and non-verbal stimuli, recorded on a compact disc device:

• Dichotic digits test: assesses figure-ground ability through the task of binaural integration. The evaluation was performed with the intensity of the stimulus to 50dBHL (average of 500Hz, 1KHz and 2KHz).
• Speech Intelligibility with Ipsilateral Competitive Message: evaluates the figure-ground skill. The intensity of 40dBHL was used (average of 500Hz, 1KHz and 2KHz).
• Consonant-Vowel Dichotic test: consists of the simultaneous presentation of syllables in both ears. The free attention stage was performed with the objective of verifying hemispheric dominance for language. The intensity of 50dBHL was used (average of 500Hz, 1KHz and 2KHz)
• Gaps-in-noise: assesses temporal resolution ability. The test was applied in the condition monaural at an intensity of 55dBHL (average of 500Hz, 1KHz and 2KHz).
• Frequency pattern test: assesses temporal ordering ability. The intensity for the test was 50 dBHL (average of 500Hz, 1KHz and 2KHz).

Auditory Brainstem Response (ABR) was performed with the purpose of verifying the integrity of the brainstem, with the presence or absence of electrophysiological waves I, III and V and the analysis of their latencies absolute and interpeaks I-III, I-V and III-V. The intensity used to assess the integrity of the auditory pathway and to analyze and compare the absolute and interpeak latencies of waves I, III and V was 80dBSPL.

Long Latency Auditory Evoked Potential (LLAEP) was performed by a two-channel device with a 1-30 Hz filter. The acoustic stimulus was monaural through earphones at 75dBSPL.

The comparison of the research groups was performed using ANOVA for ABR and P300 responses and Wilcoxon and Mann-Whitney tests for CAP responses. Gender and side were included in both models as a fixed effect, as well as their interactions. When the interaction effect between the side and group was considered significant, p-value ≤ 0.05, the ears were analyzed separately.

## Results

Participants with cystic fibrosis did not depend on oxygen therapy and had no limitations in their life activities due to the disease. They were able to attend the audiological assessment clinic to participate in the research. Among them, 41 patients were treated with daily use of inhaled aminoglycoside (tobramycin) with monthly breaks for at least 6 months and 16 underwent at least two cycles of intravenous aminoglycoside and a maximum of 4 cycles (15-day cycles) with combinations of medications amikacin, ceftazidime, sulfamethoxazole + trimethoprim and oxacillin.

Table 1 presented the tonal thresholds at frequencies from 250 to 16,000Hz obtained in the control and study groups. It shows that there was a statistically significant difference in auditory thresholds at all frequencies evaluated in the comparison between groups.

**Table 1:** Mean, standard deviation, minimum and maximum values of hearing thresholds in dBHL at frequencies from 250 to 16,000Hz, considering the groups.

Frequency (Hz)	Group	N	media	SD	minimum	maximum	p-value*
250	CG	120	5.08	4.16	-5.0	15.0	**<0.001**
	SG	114	7.54	5.07	0	25.0	
500	CG	120	4.92	3.09	-5.0	10.0	**<0.001**
	SG	114	7.23	5.23	-5.0	25.0	
1,000	CG	120	1.91	4.49	-10.0	10.0	**<0.001**
	SG	114	7.01	5.39	-5.0	25.0	
2,000	CG	120	3.25	4.02	-10.0	10.0	**<0.001**
	SG	114	7.28	5.47	-5.0	25.0	
3,000	CG	120	4.45	3.96	-10.0	20.0	**<0.001**
	SG	114	8.20	6.17	-5.0	25.0	
4,000	CG	120	3.95	4.12	-10.0	15.0	**<0.001**
	SG	114	8.37	5.56	-5.0	25.0	
6,000	CG	120	4.91	4.99	-5.0	20.0	**<0.001**
	SG	114	9.25	7.13	-5.0	35.0	
8,000	CG	120	5.12	4.47	-5.0	20.0	**<0.001**
	SG	114	9.60	5.08	0	25.0	
9,000	CG	120	1.29	5.97	-10.0	20.0	**<0.001**
	SG	114	9.95	8.06	0	55.0	
10,000	CG	120	-0.5	5.28	-10.0	15.0	**<0.001**
	SG	114	9.86	7.58	-5.0	45.0	
11,200	CG	120	0.16	6.88	-10.0	20.0	**<0.001**
	SG	114	10.30	8.45	-10.0	55.0	
12,500	CG	120	-0.08	6.82	-10,0	20.0	**<0.001**
	SG	114	12.41	8.42	0	50.0	
14,000	CG	120	-2.66	7.14	-10.0	20.0	**<0.001**
	SG	114	9.73	9.35	-5.0	50.0	
16,000	CG	120	-7.25	8.21	-10.0	10.0	**<0.001**
	SG	114	11.53	8.43	0	50.0	

Hz, Hertz; dBHL, decibel hearing level; N, number of ears; SD, standard deviation.

⁎ U of Mann-Whitney

Table 2 describes the performance in the auditory processing assessment center considering the groups. It was observed that there was a difference between the groups in the results of the gaps in the noise test. The difference between the ears regarding the CVD test reflects the left hemispheric dominance in the participants of the groups.

**Table 2:** Mean and standard deviation of the CAP behavioral tests considering the groups studied.

		CG		SG		
Tests		media	SD	media	SD	p-value
DDT		98.20%	2.61	97.64%	2.96	0.786
PPT		73.98%	20.63	75.08%	18.99	0.085
GIN		4.23ms	0.97	6.15ms	1.50	**<0.001**
SSI		69%	14.78	69%	15.77	0.843
CVD	RE	13.60	1.91	12.75	1.42	0.060
	LE	5.24	2.32	6.36	2.62	**0.034**

CG, control group; SG, study group; RE, right ear; LE, left ear; SD, standard deviation; DDT, dichotic digit test; FPT, frequency pattern test; GIN, gaps-in-noise; SSI, Speech Intelligibility with Ipsilateral Competitive Message; CVD, Consonant-Vowel Dichotic Test.

In the analysis of the results between ears for the ABR and LLAEP tests, there were no differences between the ears for both control and study groups in measures of latencies and amplitudes. For this reason, the data from the two ears were combined.

Table 3 presents the results obtained in the Auditory Brainstem Response – ABR considering the groups. There was a statistical difference between the groups in latency and amplitude of wave I and latency of wave V. In the analysis of interpeaks, a statistical difference was observed between the groups in the latencies of interpeaks I-III and III-V.

**Table 3:** Mean and standard deviation of the ABR considering the groups.

Latency (ms) Amplitude (μV)	Group	Media	SD	p-value
**Wave I**				
Latency	CG	1.66	0.11	**0.044**
	SG	1.71	1.23	
Amplitude	CG	0.46	0.11	**0.002**
	SG	0.41	0.07	
**Wave III**				
Latency	CG	3.81	0.10	0.469
	SG	3.80	0.10	
Amplitude	CG	0.51	0.12	0.915
	SG	0.52	0.08	
**Wave V**				
Latency	CG	5.59	0.14	**0.010**
	SG	5.66	0.12	
Amplitude	CG	0.74	0.20	0.376
	SG	0.72	0.08	
**Interpeak I-III**				
Latency	CG	2.14	0.15	**0.020**
	SG	2.09	0.14	
**Interpeak III-V**				
Latency	CG	1.78	0.15	**0.005**
	SG	1.86	0.14	
**Interpeak I-V**				
Latency	CG	3.96	0.17	0.347
	SG	3.95	0.16	

CG, control group; SG, study group; SD, standard deviation.

Table 4 presents the results obtained in the Long Latency Auditory Evoked Potential – PEALL research. There is a difference between the groups in the latency of the P300 wave.

**Table 4:** Mean, standard deviation, minimum and maximum values of latency (ms) and amplitude (μV) in LLAEP considering the groups.

Latency (ms) Amplitude (μV)	Group	N	media	SD	minimum	maximum	p-value*
**N1**							
Latency	CG	120	108.48	19.14	89.71	125.83	0.440
	SG	114	107.69	19.38	90.17	129.92	
Amplitude	CG	120	3.46	1.17	2.93	3.57	0.267
	SG	114	3.41	2.07	2.72	4.94	
**P2**							
Latency	CG	120	152.18	23.16	139.92	178.65	0.891
	SG	114	153.84	24.34	140.5	176.98	
Amplitude	CG	120	3.76	1.21	2.96	4.65	0.915
	SG	114	3.62	2.04	2.59	4.86	
**N2**							
Latency	CG	120	206.97	29.33	200.94	240.91	0.315
	SG	114	209.43	30.21	204.83	269.93	
Amplitude	CG	120	4.74	2.40	3.26	5.76	0.376
	SG	114	4.52	2.89	3.39	5.78	
**P300**							
Latency	CG	120	318.95	30.64	292.49	367.15	**<0.001**
	SG	114	327.86	38.29	300.35	380.43	
Amplitude	CG	120	5.44	2.50	4.12	6.23	0.079
	SG	114	5.37	2.08	4.01	6.49	

CG, control group; SG, study group; SD, standard deviation; N, number of ears.

⁎ ANOVA.

## Discussion

All participants had previously undergone peripheral hearing assessment and presented results within the normal range, according to the WHO classification.

The findings in the peripheral hearing assessment (pure tone audiometry) are consistent with normality when using the World Health Organization classification.^20^ It was found, however, that there was a statistically significant difference when compared with the control group. The same happens in high-frequency audiometry, in all frequencies evaluated (Table 1). These findings may reflect in a way that has not yet been established in the literature in the findings of the central auditory pathway, as the stimulus is influenced by the entire course of the auditory pathway, from its most peripheral portions to the auditory cortex.

Central Auditory Processing (CAP) consists of a complex activity that involves neurophysiological events that provide the ability to use sound information using auditory skills. This activity starts in the cochlea and progresses to the auditory cortex.

In the study, the intensities used for the behavioral assessment of CAP are those validated for each procedure, so that the authors could use the normality criteria validated by the authors of each test.

There was no statistically significant difference between the right and left ears in the CG and SG with regard to the DDT, FPT, GIN and SSI tests. The difference found between the ears refers to the CVD test, due to the left hemispherical predominance.

The CVD was performed in this study in order to verify left hemispheric dominance in all participants since right-hand preference was not an inclusion criterion in the study. Thus, with the CVD result indicating a predominance of responses in the right ear in the free attention stage, the authors can verify that the linguistic hemispheric predominance in all participants was on the left side.

The results without statistical difference between the ears were combined, and when comparing the results between CG and SG, a statistically significant difference was found in the results of the GIN test, with a mean gap in the CG of 4.23ms and 6.15ms in the SG (Table 2).

Temporal auditory processing can be divided into four categories. They are: ordering, integration, masking and resolution. The GIN concerns the temporal resolution category, which allows transient acoustic changes to be identified. This category is essential for language skills and reading activities.

There are studies that suggest that temporal auditory processing may be compromised in sensorineural hearing loss, which is not widely accepted. However, there seems to be a relationship between an increase in the gap threshold in the test and greater hearing loss at high frequencies.^23^ This relationship is important in the findings of the investigation of patients with CF, since the hearing alterations characteristic of patients exposed to AG are of the sensorineural type and affect the higher auditory frequencies earlier.

ABR is a potential that assesses auditory functioning at the brainstem level. It is known that the auditory system is already formed in utero and its maturation process takes place until the first years of life. Therefore, the latency results found in the brainstem potentials have a decrease along the process.

In the present study, no difference was observed between the right and left ears in the control and study groups. Results were grouped between ears.

The measurements of absolute and interpeak latencies in both groups are within the expected values, according to the standardization of the equipment used. This result proves the integrity of the brainstem. However, a statistically significant difference was found in the latency responses in waves I and V in the comparison between the CG and SG, with higher latencies in the SG (Table 3). The results of the peripheral evaluation were normal in the frequency range that includes the stimulus used to evoke brainstem-evoked potentials. The fact that the difference between the groups did not appear in wave III encourages us to further investigate how the difference found when comparing the groups in audiometry can be related to the ABR findings.

The difference between groups in ABR was not maintained in waves N1, P2, N2. The lack of influence of short latency waves on medium latency potentials has already been described^24^ and appears to be related to different stimuli that can be used for evaluations and the fact that these potentials are less dependent on neural synchrony than the ABR. The characteristic of the stimulus may be closely related to this finding and the same may happen for long latencies.

A difference was also found between latencies in the interpeak intervals I-III and III-V. In the I-III interval, the latencies were higher in the CG, different from the III-V interval in which the higher latencies are found in the SG (Table 3).

The analyzed data show a significant difference in the amplitude of wave I in the comparison between CG and SG, with lower amplitudes in the SG. No difference was found in the I-V interpeak interval between the groups (Table 3).

In a previous study, latency and amplitude changes were not found in the ABR of patients with cystic fibrosis.^25^ The same study verified preserved peripheral hearing and suggested that neither the disease itself nor the treatment performed with potentially ototoxic drugs interfered with the auditory system of the population evaluated.

LLAEP can inform about some superior functions of the SNAC, such as categorization functions, ability to perceive stimulus duration and attention to sound information.

The LLAEP analysis between the CG and SG shows a statistically significant difference in the latency of the P300 potential, with higher latencies in the SG (Table 4).

This potential is closely related to the capacity for attention. No difference was found between the groups regarding the N1, P2 and N2 potentials and in the measure of the amplitude of the potentials between the groups.

No studies were found in the literature associating CF with LLAEP. Most of the studies carried out use LLAEP to assess children with cognitive, attention, CAP disorders and learning difficulties, complaints not frequently related to patients with CF.

The significant differences between the increase in latency of wave I and interpeak III-V and the reduction of wave I in the EG may be due to the reduced number of nerve fibers stimulated during the presentation of the auditory stimulus and to a possible decrease in the speed of information. These characteristics may be due to a lack of auditory stimulation.

This study differs from others by bringing to light the characterization of the central portion of hearing in participants with cystic fibrosis, as the scientific literature widely addresses the consequences of medications on the peripheral portion of hearing.^26^, ^27^, ^28^ However, the logistics of the study end up involving, unintentionally, patients with less severity of the disease coming to the clinic for hearing assessment. The authors strongly recommend that further studies be carried out and that in other studies the sample of patients is diverse in terms of the severity of the disease and that the use of medications can be monitored more precisely so that the investigation of the central auditory system is more enlightening.

The authors suggest with this study the importance of monitoring not only the functions performed by the peripheral auditory system, but also the functions of the central auditory system so that there is an early, specific, and individualized action in cases where changes are observed that compromise the ability and quality of understanding sound information.

Children and adolescents with cystic fibrosis performed worse of the gaps-in-noise test to assess Central Auditory Processing when compared to the CG, which indicates impairment of the temporal auditory ability.

They also showed increased latency in ABR waves I and V, in addition to increased P300 latency in LLAEP.

## Conflicts of interest

The authors declare no conflicts of interest.
